# A Novel Risk Stratification System for Thyroid Nodules With Indeterminate Cytology—A Pilot Cohort Study

**DOI:** 10.3389/fendo.2020.00053

**Published:** 2020-02-18

**Authors:** Cristiane J. Gomes-Lima, Sungyoung Auh, Shilpa Thakur, Marina Zemskova, Craig Cochran, Roxanne Merkel, Armando C. Filie, Mark Raffeld, Snehal B. Patel, Liqiang Xi, Leonard Wartofsky, Kenneth D. Burman, Joanna Klubo-Gwiezdzinska

**Affiliations:** ^1^National Institutes of Health/National Institute of Diabetes and Digestive and Kidney Diseases (NIH/NIDDK), Bethesda, MD, United States; ^2^MedStar Clinical Research Center, MedStar Health Research Institute (MHRI), Washington, DC, United States; ^3^National Institutes of Health/National Cancer Institute (NIH/NCI), Bethesda, MD, United States; ^4^Division of Endocrinology, Department of Medicine Georgetown University, Washington, DC, United States

**Keywords:** thyroid nodule, thyroid ultrasound, fine needle aspiration biopsy, indeterminate cytology, molecular testing

## Abstract

**Background:** Thyroid ultrasound (US), fine needle aspiration biopsy (FNAB), and molecular testing have been widely used to stratify the risk of malignancy in thyroid nodules. The goal of this study was to investigate a novel diagnostic approach for cytologically indeterminate thyroid nodules (ITN) based upon a combination of US features and genetic alterations.

**Methods:** We performed a pilot cohort study of patients with ITN (Bethesda III/IV), who underwent surgical treatment. Based on standardized sonographic patterns established by the American Thyroid Association (ATA), each ITN received an US score (X_US_), ranging between 0 and 0.9 according to its risk of thyroid cancer (TC). DNA and RNA were extracted from pathologic material, available for all patients, and subjected to Oncomine™ Comprehensive Assay v2 (OCAv2) next-generation sequencing. Each genetic alteration was annotated based on its strength of association with TC and its sum served as the genomic classifier score (X_GC_). The total risk score (TRS) was the sum of X_US_ and X_GC_. ROC curves were generated to assess the diagnostic accuracy of X_US_, X_GC_, and TRS.

**Results:** The study cohort consisted of 50 patients (39 females and 11 males), aged 47.5 ± 14.8 years. Three patients were excluded due to molecular testing failure. Among the remaining 47 patients, 28 (59.6%) were diagnosed with TC. *BRAFV600E* was the most common mutation in papillary TC, *PAX8-PPARG* fusion was present in NIFTP, pathogenic variants of *SLX4, ATM*, and *NRAS* were found in Hürthle cell TC and *RET* mutations in medullary TC. The diagnostic accuracy of X_GC_ and TRS was significantly higher compared with X_US_ (88 vs. 62.5%, *p* < 0.001; 85.2 vs. 62.5%, *p* < 0.001, respectively). However, this increased accuracy was due to significantly better sensitivity (80.7 vs. 34.6%, *p* < 0.001; 84.6 vs. 34.6%, *p* < 0.001, respectively) without improved specificity (94.7 vs. 90%, *p* = 0.55; 85.7 vs. 90%, *p* = 0.63, respectively).

**Conclusion:** Molecular testing might not be necessary in ITN with high-risk US pattern (X_US_ = 0.9), as specificity of TC diagnosis based on Xus alone is sufficient and not improved with molecular testing. OCAv2 is useful in guiding the management of ITN with low-to-intermediate risk US features (X_US_ < 0.9), as it increases the accuracy of TC diagnosis.

## Introduction

The management of patients with thyroid nodules has evolved with the widespread use of the Bethesda System for Reporting Thyroid Cytopathology (1). Although the approach is standardized, especially for nodules with clearly benign (Bethesda category II) or malignant features (Bethesda category VI), the management of patients with thyroid nodules yielding an indeterminate cytology [Bethesda III—atypia of unknown significance (AUS) or follicular lesion of undetermined significance (FLUS); Bethesda IV—follicular neoplasm (FN) or suspicious for a follicular neoplasm (SFN)], which account for 10–25% of thyroid fine needle aspiration biopsies (FNABs) is still very challenging (1, 2). The current ATA guidelines recommend either surveillance or diagnostic surgery for nodules in category III and surgical excision for nodules in category IV, in which molecular testing was not performed or was inconclusive (3). The standardization of malignancy risk stratification based on ultrasound (US) imaging of the neck has been recently proposed by several organizations. American and European Thyroid Associations have implemented representative pictorial systems based on sonographic patterns of thyroid nodules (3, 4), while the Korean Society of Thyroid Radiology and the American College of Radiologists utilize a scoring system—K-TIRADS and TIRADS (5, 6). These systems are designed to standardize the management strategy and enable easier communication between patients and endocrinologists, radiologists and surgeons (3). Recently established machine learning algorithms, used to characterize the US patterns typical of malignancy, have been also successfully implemented (7–9).

Molecular testing has been evolving as a useful tool in guiding the decision-making regarding surgical vs. conservative management of cytologically indeterminate thyroid nodules (10–13). From single gene mutation testing to next generation sequencing (NGS) multigene panels, as well as gene expression classifiers and microRNA (miRNA) markers, the available diagnostic techniques vary in sensitivity and specificity (14–21).

Despite advances in sonographic systems to classify thyroid nodules according to their risk of malignancy and the added potential utility of molecular diagnosis, overtreatment of thyroid nodules is still commonly observed and associated with substantial side effects and incremental cost (11).

A holistic approach to the patient with an indeterminate thyroid nodule, incorporating clinical, sonographic, cytological, and molecular data is optimal in decision making process. Thus, the goal of this study was to investigate the diagnostic accuracy of a risk stratification system for cytologically indeterminate thyroid nodules based on a combination of US features and genetic abnormalities.

## Methods

We performed a single-institution pilot cohort retrospective study including patients with thyroid nodules who underwent thyroid US and FNAB revealing indeterminate cytology (Bethesda III, IV), and were subsequently subjected to surgical treatment. These patients presented in the Thyroid Outpatient Clinic and the decision to perform FNAB was based on the current ATA guidelines (3). However, there were 2 patients for whom nodules <1 cm were biopsied—one due to cervical lymphadenopathy and one due to family history of papillary thyroid cancer in multiple family members. In the presence of indeterminate cytology, patients were offered either surgery or follow-up based on clinical and ultrasonographic criteria during subsequent visits. Only patients with cytologically indeterminate nodules that had surgery were included in this analysis.

We excluded patients characterized by Bethesda I, II, IV, VI cytology categories and subjects who have not been treated with surgery for indeterminate thyroid nodules.

The study was approved by the NIH Intramural Institutional Review Board. Informed consent has been obtained from each patient after full explanation of the purpose and nature of all procedures performed.

### Evaluation of US Patterns

Based on American Thyroid Association (ATA) sonographic patterns, each nodule received an annotated ultrasound score (X_US_), according to its risk of malignancy with 0 for benign and very low suspicion pattern (<3% cancer risk per the ATA guidelines), 0.1 for low suspicion (5–10% malignancy risk per the ATA guidelines), 0.2 for intermediate suspicion (10–20% malignancy risk per the ATA guidelines), and 0.9 for high suspicion nodules (>70–90% cancer risk per the ATA guidelines) (3). In other words, numbers of X_US_ refer to the percentages of the upper limit of malignancy per ATA guidelines: 0.1 = 10% (low risk); 0.2 = 20% (intermediate risk); 0.9 = 90% (high risk). Two endocrinologists specialized in thyroid disorders (CG-L and JK-G) reviewed independently the entire registered trailers of thyroid and neck US and were blinded to pathology and molecular test results. The discordance between the evaluation of the nodules was addressed by reviewing the nodules again with a third party—radiologist, until consensus was met.

### Evaluation of the Molecular Signature of Thyroid Nodules

DNA and RNA were extracted from formalin-fixed paraffin embedded tumors obtained at surgery. Next generation sequencing was performed using Oncomine^TM^ Comprehensive Assay v2 (OCAv2) on an Ion Torrent S5 XL sequencer. The Oncomine™ Comprehensive Assay v2 (OCAv2) is a CLIA (Clinical Laboratory Improvement Amendments)-validated commercial pan-cancer targeted NGS panel designed to detect somatic single-nucleotide variants (SNV), insertions and deletions (INDEL), copy number variants (CNV), and gene fusions in 143 genes, including major driver genes in thyroid oncogenesis (Supplemental Figure 1). OCAv2 utilizes Ion AmpliSeq^TM^ chemistry, allowing for DNA and RNA inputs as low as 10 ng of extracted material from formalin-fixed paraffin embedded (FFPE) tumor samples. The variant calling, annotation, and classification were performed on Ion Reporter software v5.10 (Thermo Fisher). In addition, a droplet digital PCR was performed to assess TERT promoter mutation. TERT promoter c.1-124C>T and c.1-146C>T mutational analysis were performed using the expert design PrimePCR ddPCR TERT C228T_113 Assay and TERT C250T_113 Assay (BIO-RAD, Hercules, CA) on a BIO-RAD QX200 droplet digital PCR (ddPCR) system. The assays were performed in duplicate. The presence of mutation and the fractional abundance of the mutant allele were determined using QuantaSoft v.1.7 (BIO-RAD).

The dataset of ultrasound/FNAB was cross-checked with the dataset of tissue molecular analysis to ensure the identity between biopsied and genotyped nodules. Each nodule received an annotated genomic classifier score (X_GC_) based on the probability of a given genomic abnormality being associated with cancer per large molecular databases TCGA and COSMIC v87. Each genomic alteration received a score ranging from 0 to 1, with 0—for no association with cancer and 1—for 100% association with cancer. Genetic abnormalities observed in both benign and malignant lesions received scores between 0 and 1 based on a ratio of their prevalence in malignant and benign lesions. The final genomic classifier (X_GC_) score was a sum of all genomic abnormalities given by the formula:

(1) $$\begin{array}{l} {\text{X}}_{\text{GC}}=\left({\text{X}}_{\text{SNV}/\text{INDEL}}\right)\text{n}+{\text{X}}_{\text{GF}}+\;{\text{X}}_{\text{CNV}} \end{array}$$

where X is the annotated score; SNV, single nucleotide variant; INDEL, insertions/deletions; *n*, number of SNVs/INDELs; GF, gene fusions; CNV, copy number variants (20).

### Risk Stratification Based on Imaging and Molecular Characteristics

A third score, called total risk score (TRS), was calculated as the sum of the ultrasound score (X_US_) and the genomic classifier score (X_GC_):

(2) $$\begin{array}{l} \text{TRS}:{\text{X}}_{\text{US}}+\;{\text{X}}_{\text{GC}} \end{array}$$

## Statistical Analysis

Receiver operating characteristic (ROC) curves were generated to assess the diagnostic accuracy of X_US_, X_GC_, and TRS. First, in each scoring system, area under the curve (AUC) values were obtained with different cutoff values for dichotomization between benign and malignant nodules and the best cutoff value with the highest AUC was selected. Second, we compared the three best scoring systems by using AUC, sensitivity, specificity, positive predictive value (PPV) and negative predictive value (NPV) with 95% Wilson confidence intervals (CI). Given that the cancer prevalence may vary in different populations with cytologically indeterminate thyroid nodules, the established sensitivity and specificity were used to calculate PPV and NPV corresponding to the whole spectrum of cancer prevalence utilizing Bayes theorem using R software. All other analyses were based on two-tailed tests using α = 0.05 and conducted using SAS version 9.4 (SAS Institute, Cary, NC, USA).

## Results

**Figure 1:**
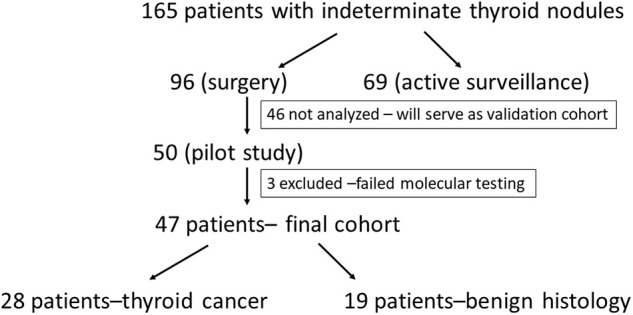
Outline of the process leading to inclusion of the patients for this pilot cohort.

Among 165 patients with cytologically indeterminate thyroid nodules being currently followed by the National Institutes of Health endocrine service, 96 patients had surgery performed prior to December 2017. We present the results of the first 50 patients (39 females and 11 males) with 87 nodules, for whom a comprehensive molecular analysis of thyroid nodules was performed. Three patients were excluded from further analysis due to insufficient quality of the RNA extracted from the pathology material (Figure 1). The mean age at the time of diagnosis was 47.5 ± 14.8 years (Table 1). The analysis of US patterns of thyroid nodules in the cohort revealed an interobserver agreement rate of 94%. The annotated ultrasound scores (X_US_) were 0.9 in 15 nodules, 0.2 in 20 nodules and 0.1 in 15 nodules. Twenty-eight patients (59.6%) were diagnosed with thyroid carcinoma (TC): 15 papillary TC (PTC) (including 4 micro-PTCs identified within biopsied nodule), 6 follicular variant of PTC (PTCFV), 1 non-invasive follicular thyroid neoplasm with papillary-like nuclear features (NIFTP), 2 Hürthle cell TC (HTC), 1 poorly differentiated TC (PDTC), and 3 medullary TC (MTC) (Table 2). Incidentally discovered microcarcinomas within the normal parenchyma, observed in 4 patients, were not considered malignant.

**Table 1:** Baseline characteristics of the study cohort.

**Clinical characteristics**	
Average ± SD age at diagnosis (years)	47.5 ± 14.8
Gender (% female)	37/47 (78.7%)
Average ± SD tumor size (cm)	2.3 ± 1.7
Surgical procedure	
Total thyroidectomy	26/47 (55.3%)
Lobectomy	21/47 (44.7%)

**Table 2:** Histology of 47 cytologically indeterminate thyroid nodules.

	***n***	**%**
**Carcinoma**	**28**	**59.6**
PTC Classic PTCFV Microcarcinoma	21 11 6 4	75.0
NIFTP	1	3.6
HTC	2	7.1
PDTC	1	3.6
MTC	3	10.7
**Benign**	**19**	**40.4**
Follicular adenoma	6	31.6
Adenomatoid nodule	13	68.4

*PTC, papillary thyroid carcinoma; PTCFV, papillary thyroid carcinoma follicular variant; NIFTP, non-invasive follicular thyroid neoplasm with papillary-like nuclear features; HTC, Hürthle cell thyroid carcinoma; PDTC, poorly differentiated thyroid carcinoma; MTC, medullary thyroid carcinoma*.

**Figure 2:**
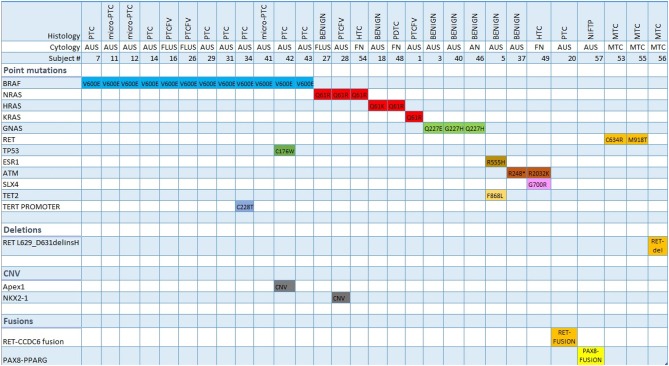
Molecular signature (Oncoprint) of indeterminate thyroid nodules tested positive by Oncomine v2 assay. PTC, papillary thyroid carcinoma; PTCFV, papillary thyroid carcinoma follicular variant; NIFTP, non-invasive follicular thyroid neoplasm with papillary-like nuclear features; HTC, Hürthle cell thyroid carcinoma; PDTC, poorly differentiated thyroid carcinoma; MTC, medullary thyroid carcinoma; AUS, atypia of unknown significance; FLUS, follicular lesion of undetermined significance; FN, follicular neoplasm; AN, adenomatoid nodule; CNV, copy number variation.

The OCAv2 testing was positive in 22/28 (78.5%) TC nodules and 7/19 (36.8%) benign nodules (Figure 2). The most frequent genetic alteration in TC, observed in 12/28 (42.8%) of patients, was BRAF p.Val600Glu mutation, present in PTC (including micro-PTC), and PTCFV, followed by NRAS and KRAS mutations in 4/28 (14.3%) of TC patients (PTCFV, PDTC and HTC). RET point mutations (p.Cys634Arg and p.Met918Thr) and a RET deletion were found in cases of MTC. HTC was characterized by the presence of NRAS, SLX4 and ATM pathogenic variants, while NIFTP by PAX8-PPARG fusion. There were 4 patients with more than one mutation present, including one patient with PTC characterized by concomitant presence of BRAF p.Val600Glu and a TERT promoter mutation (c.1-124C>T) (Figure 2).

Benign nodules were characterized by having either no genetic alteration or the presence of RAS, GNAS, ESR1, ATM, and TET2 variants (Figure 2).

### Malignancy Risk Stratification System

#### Optimal Cut-Off Value Discriminating Between Benign and Malignant Lesions Based on the Ultrasound Score (X_US_)

**Figure 3:**
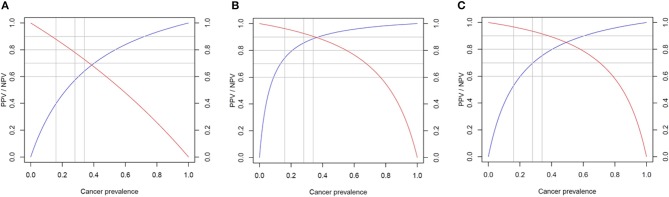
Predicted performance of each score in populations with different cancer prevalence. Negative predictive value (NPV) in red and positive predictive value (PPV) in blue. **(A)** Ultrasound score (X_US_); **(B)** genomic classifier score (X_GC_); and **(C)** total risk score (TRS).

There were no significant differences in diagnostic accuracy determined by AUC between different cutoff points for X_US_ (Supplemental Figure 2A). However, an X_US_ cutoff value of 0.9 was selected due to much higher specificity compared to a cutoff value of 0.2 (90 vs. 52.4% *p* = 0.006). The sensitivity of the US cutoff value of 0.9 was 34.6%, specificity 90%, PPV 81.8% (CI 52.3–97.9%), NPV 52.8% (CI 37–68%). Given a high prevalence of thyroid cancer in our cohort, suggesting a referral and selection bias, Bayes' theorem was used to predict NPV and PPV in populations with different cancer prevalence ranging from 6 to 59% (Figure 3). The NPV and PPV for X_US_ ranged between 52.8–95% and 18–81.8%, respectively.

#### Optimal Cutoff Value to Discriminate Between Benign and Malignant Lesions Based on the Genomic Classifier Score (X_GC_)

The X_GC_ cut off value of 0.6 was characterized by the highest diagnostic accuracy of 88% (Supplemental Figure 2B). The sensitivity of X_GC_ of 0.6 was 80.7%, specificity 94.7%, PPV 92.3% (CI 78.2–99.2%), NPV 80% (CI 60.1–91.1%). Based on Bayes' theorem prediction, the NPV and PPV for X_GC_ may range between 83–99% and 43–93%, respectively in different populations (Figure 3).

**Figure 4:**
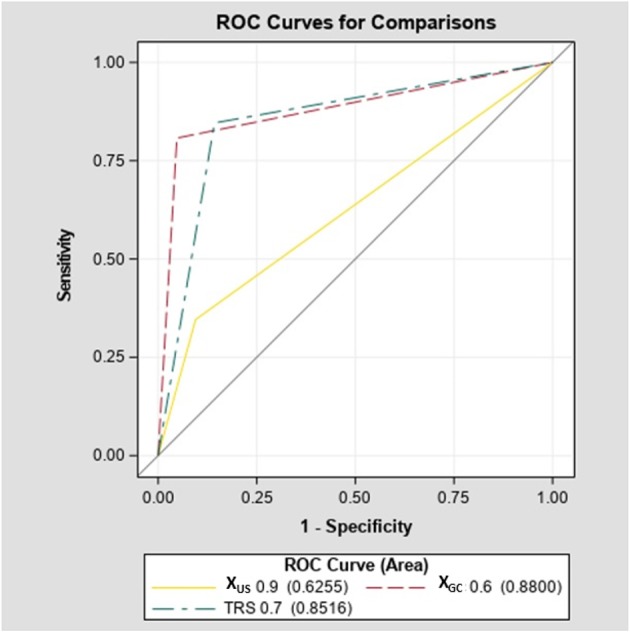
Comparison of ROC curves-diagnostic accuracy of X_US_, X_GC_, and TRS.

The diagnostic accuracy of X_GC_ of 0.6 was significantly higher compared with X_US_ of 0.9 (88 vs. 62.5%, *p* < 0.001; Figure 4). However, an increased accuracy was due to significantly better sensitivity (80.7 vs. 34.6%, *p* < 0.001) without further improvement is specificity (94.7 vs. 90%, *p* = 0.55).

#### Optimal Cutoff Value to Discriminate Between Benign and Malignant Lesions Based on Total Risk Score (TRS)

The TRS based cutoff of 0.7 was associated with the highest diagnostic accuracy of 85.2% (Supplemental Figure 2C). The sensitivity of TRS 0.7 was 84.6%, specificity 85.7%, PPV 88% (CI 70–95.8%), NPV 81.8% (CI 61.5–92.7%). The NPV and PPV in populations characterized by different cancer prevalence is predicted to range between 82–99% and 27–88%, respectively (Figure 3).

The diagnostic accuracy of TRS of 0.7 was significantly higher compared with the X_US_ of 0.9 (85.2 vs. 62.5%, *p* < 0.001; Figure 4). However, the increased accuracy was due to significantly better sensitivity (84.6 vs. 34.6%, *p* < 0.001) without further improvement in specificity (85.7 vs. 90%, *p* = 0.63). There was no difference in diagnostic accuracy between TRS and X_GC_ (85.2 vs. 88%, *p* = 0.46; Figure 4). In addition, in a sub-analysis of our cohort excluding patients with MTC, the sensitivity of X_GC_ was 78.2%, specificity of 95.2% while the sensitivity of TRS was 82.6% and specificity of 85.7% (Supplemental Figures 4–6).

Given a high and non-inferior to molecular testing specificity of US alone for US high-risk nodules, we next performed a subgroup analysis testing the diagnostic accuracy of OCAv2 in group A—US high-risk nodules and group B—US low-to-intermediate risk nodules.

### Diagnostic Algorithm for Management of Thyroid Nodules

Patients with X_US_ of 0.9 were characterized by a 5x higher likelihood of cancer than the patients with X_US_ < 0.9 (OR 5.03, 95%CI 0.95–26.6). The subgroup analysis of 11 patients with a X_US_ of 0.9 revealed that implementing X_GC_ or TRS does not lead to improved diagnostic accuracy, as X_US_ alone led to a comparable separation of benign vs. malignant lesions (Supplemental Figure 3A). In contrast, molecular tests significantly improved diagnostic accuracy in patients with X_US_ < 0.9 (Supplemental Figure 3B). Diagnostic accuracy of X_US_ of 0.2 was 61%—significantly lower than 82.7% of X_GC_ (*p* = 0.04) and 85.6% of TRS (*p* = 0.01; Supplemental Figure 3B). X_GC_ and TRS significantly improved specificity of cancer diagnosis in this group (X_GC_ vs. X_US_ 94.7 vs. 57.9%, *p* = 0.007 and TRS 94.7 vs. 57.9%, *p* = 0.007), without a significant improvement in sensitivity (X_GC_ vs. X_US_ 70.6 vs. 64.7%, *p* = 0.71 and TRS vs. X_US_ 76.5 vs. 64.7, *p* = 0.45). There was no significant difference in diagnostic accuracy between X_GC_ and TRS (82.7 vs. 85.6%, *p* = 0.32). However, if the analysis had been performed prospectively, TRS < 0.7 could have led to missing diagnosis of cancer in 4 very low risk tumors—two mutation negative FVPTC T1bN0M0, both with X_US_ of 0.2 consistent with intermediate features per the US characteristics, one mutation negative classic PTC T1bN0M0 characterized by X_US_ of 0.1 consistent with low risk US features, and one mutation negative micro-PTC T1aN0M0, with X_US_ of 0.1, multifocal, with the maximum diameter of the largest lesion of 0.8 cm—while X_GC_ < 0.6 could have led to missing cancer diagnosis not only in the above mentioned 4 very low risk tumors, but also in one PDTC with *HRASQ61R* mutation, characterized by intermediate risk US features with X_US_ of 0.2. Altogether, this suggests that TRS may have a better clinical performance.

**Figure 5:**
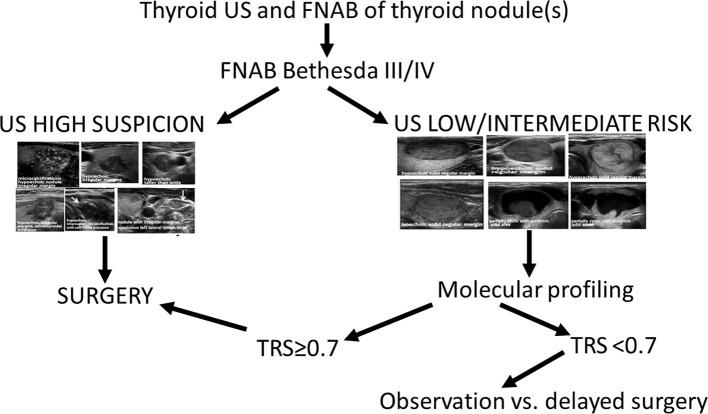
Proposed algorithm for diagnosis and management of cytologically indeterminate nodules.

Based on this analysis, we propose the following algorithm for the management of thyroid nodules with indeterminate cytology (Figure 5).

1. X_US_ high risk = 0.9—no added benefit in specificity of molecular testing—consider referral for surgical treatment
2. X_US_ < 0.9 (low to intermediate risk nodules)—diagnostic accuracy improves with molecular testing—consider surgery if TRS ≥ 0.7; if TRS < 0.7, a watchful waiting strategy with observation of tumor behavior over time might be a reasonable option as all cancers that could have been missed in this strategy were very low risk micro-PTCs.

## Discussion

We propose a novel diagnostic algorithm for patients with Bethesda III/IV cytology diagnosis, based on a combination of US patterns and the molecular signature of thyroid nodules. We show that OCAv2 molecular profiling is not associated with a significant improvement in specificity of cancer diagnosis in cytologically indeterminate thyroid nodules characterized by high-risk US features. However, OCAv2 molecular profiling is useful in improving diagnostic accuracy of cytologically indeterminate thyroid nodules characterized by low-to-intermediate sonographic features. Therefore, we propose a sequential approach for patients with AUS/FLUS/FN/SFN cytology diagnosis that consists of:

1. Reviewing patients' US images and considering surgical treatment for those with ATA high-risk US features nodules, without further molecular profiling, as the US alone-based specificity of cancer diagnosis in these nodules is non-inferior to molecular profiling;
2. performing molecular profiling for US low-to-intermediate risk nodules and considering surgical treatment for patients characterized by molecular score TRS of ≥0.7 and watchful waiting strategy with an observation of tumor growth over time as a reasonable option for molecular score TRS < 0.7.

This approach should be associated with an improved cost to benefit ratio, as routine implementation of molecular diagnostics is expensive. Costs of molecular testing vary according to insurance coverage but reported costs for the most comprehensive tests range between $3,000–$4,800 per nodule (12, 13). That being said, some centers may utilize high-risk molecular signature (e.g., concomitant *BRAFV600E* and *TERT* promoter mutation, EIF1AX mutations and THADA fusions) to guide the extend of surgery, in which case analysis of molecular signature of all nodules is warranted.

Several studies have suggested that sonographic patterns can effectively stratify the prevalence of malignancy in indeterminate thyroid nodules (22–25). A retrospective study of 173 indeterminate thyroid nodules reported a cancer rate of 75% in high suspicion nodules compared to 16.8% in very low, low and intermediate suspicion nodules, per ATA US patterns (24). Another retrospective study, also using ATA US patterns, including 463 indeterminate thyroid nodules found 5 times higher likelihood of malignancy in high risk patterns compared with low-to-intermediate risk patterns (25).

However, the appropriate risk stratification of thyroid nodules based on US patterns depends on the experience of the ultrasonographer and the characteristics of the equipment. Choi et al. compared interobserver and intraobserver variations in ultrasound assessment of thyroid nodules and concluded that the final assessments of experienced radiologists were highly accurate (26). These results were corroborated by another study that demonstrated that trainees receiving one-on-one instruction from experienced radiologists improved their diagnostic performance for evaluating thyroid nodules with ultrasonography (27).

The use of molecular markers for cytologically indeterminate thyroid nodules has been evolving over the past two decades (11–13). We used the Oncomine™ Comprehensive Assay v2 (OCAv2), a pan-cancer 143-gene panel focused on potentially actionable oncogenes relevant in precision medicine. We created a scoring system based on the strength of association with cancer ranging from 0 (no association with cancer) to 1 (100% association with cancer). Similarly, ThyroSeq v3, a 112-gene panel, uses a genomic classifier score in which each detected genetic alteration receives a value from 0 to 2 based on the strength of its association with malignancy (20, 21). In Bethesda III and IV nodules combined, the ThyroSeq v3 demonstrated a 94% sensitivity and 82% specificity (21). In our study, the X_GC_ was characterized by lower than ThyroSeqv3 sensitivity of 80.7%, but higher specificity of 94.7%. The lower sensitivity is most likely because Thyroseqv3, as a thyroid specific gene panel, consists of an analysis of common as well as very rare mutations observed in thyroid cancer, while OCAv2 has a broader spectrum of pan-cancer oncogenes, and is covering only the most common mutations associated with thyroid cancer. Therefore, while with proposed by us approach there might be a higher risk of missing thyroid cancer amongst indeterminate thyroid nodules due to lower sensitivity compared with Thyroseq v3, its high specificity may help with avoiding unnecessary surgeries.

Another widely utilized diagnostic approach is the Afirma assay by Veracyte, Inc. It is based on the use of messenger-RNA (mRNA) expression and a proprietary machine learning algorithm to classify the risk of malignancy of a given nodule into benign or suspicious. The Afirma Gene Expression Classifier (GEC) was validated in a large cohort of patients in 2012 as a relatively good rule-out test with high sensitivity and a NPV from 75 to 100% (14). However, post-validation studies have shown that Afirma GEC did not perform as expected on Hürthle cell-rich lesions and had a lower than anticipated malignancy rate within GEC-suspicious nodules (28–30). An updated version of Afirma called Genomic Sequencing Classifier (GSC) was validated in 2018 with a reported sensitivity of 91% and a specificity of 68% (31). An independent comparison between Afirma GEC and GSC showed that the latter version has a higher benign call rate compared to the former, predominantly for Hürthle cell cytology (32). These findings were recently corroborated by another study, from a single academic tertiary center, that also reported an improvement in specificity and PPV of Afirma GSC, while maintaining high sensitivity and NPV (33). Compared with Afirma, OCAv2 is characterized by lower sensitivity of 84.6%, but higher specificity of 85.7%.

ThyGeNEXT/ThyraMIR, previously known as ThyGenX/ThyraMIR, is a next generation sequencing test for mutations (10 genes) and fusions (6 genes) implicated in thyroid tumorigenesis complemented with expression analysis of 10 microRNAs (miRNA). It uses a proprietary algorithm to classify each nodule as having a high risk or low risk miRNA profile (12). This combined algorithm achieved a sensitivity of 89% and a specificity of 85% among cytologically indeterminate nodules in a cross-sectional study (18). No post-validation studies have been reported. Compared with ThyGeNEXT/ThyraMIR, OCAv2 is characterized by similar sensitivity and specificity.

While sensitivity and specificity of a diagnostic test depend on test performance, negative predictive value (NPV) and positive predictive value (PPV) depend on the prevalence of disease in the population. Our study was most likely associated with a referral and selection bias, as the prevalence of cancer in our sample of 50 patients was as high as 59%. Analysis of all 96 patients who underwent surgery revealed cancer prevalence of 45.8% (44/96) and assuming that non-operated patients were characterized by benign disease, cancer prevalence would have been 26.6% (44/165) (Figure 1). The analysis of two recent reports testing performance of Afirma GSC amongst patients with indeterminate thyroid nodules, who underwent surgery, revealed a similar cancer prevalence of 50–55% (32, 33). The TRS-based NPV of 82% is significantly lower than reported in these studies NPV of 100%, while TRS-based PPV of 88% is significantly better than reported in these studies PPV of 50–60% (32, 33). Compared with Afirma GSC, in populations with cancer prevalence 50–59%, TRS may perform better in avoiding surgery for benign nodules but might be associated with higher risk of missing cancer. That being said, implementing our approach prospectively would have led to 4 missed cases of cancer in our cohort—all very low risk microcarcinomas. We have also performed a Bayes' theorem-based simulation, documenting that NPV and PPV of TRS in populations with cancer prevalence of 6–59% would range from 82 to 99% and 27–88%, respectively. In particular, comparing with Thyroseq v3 tested on a population with 28% cancer prevalence (21), TRS may perform similarly—as Thyroseq v3 with NPV of 97% performs slightly better than predicted TRS-based NPV of 93.4% while Thyroseq v3 PPV of 66% is slightly worse than predicted TRS-based PPV of 69.7% (Figure 3). The performance of ThyGeNEXT/ThyraMir in a population with cancer prevalence of 32% is very similar to predicted TRS performance—NPV of 94 vs. 93% and PPV of 74 vs. 72%, respectively [Figure 3; (18)]. Obviously, only head to head comparisons in well-designed non-inferiority trials would enable reaching any conclusions about the accuracy of the above-mentioned tests, as the comparison performed above is based on simulation rather than actual hard data. The strengths of our study rely on broad clinical data and pathologic diagnosis available for all patients. All ultrasound studies were performed with the same equipment and reported by board-certified radiologists and independently reviewed by two endocrinologists, blinded to histological diagnosis. All cytological and histological diagnosis were made by board-certified and experienced pathologists. Moreover, Oncomine™ is a widely available assay used by many molecular diagnostics laboratories. In addition, the algorithm proposed, utilizing a combination of US features and molecular diagnostics in malignancy risk stratification of thyroid nodules, might be applicable to any other molecular test available worldwide in different institutions.

We do acknowledge a significant referral and selection bias in our cohort as a potential limitation of this study. Some patients were referred to our institution with the intent of having surgery. Moreover, as a retrospective study, we could not control for factors that prompted certain patients for surgery instead of conservative management. Yet, we do provide a simulation of the performance of X_US_, X_GC_, and TRS in cohorts with different cancer prevalence according to the Bayes theorem, with ranges of NPV and PPV for each score (Figure 3).

Moreover, this pilot study is limited by the small sample size and reduced number of Hürthle cell carcinomas, NIFTP and other follicular architecture tumors. The nomenclature revision of encapsulated follicular variant of PTC (EFVPTC) to non-invasive follicular thyroid neoplasm with papillary-like nuclear features (NIFTP) (34) may represent the acceptance of borderline/precursor lesions in the thyroid (35); additional information is warranted about the molecular signature of these tumors. The relative high prevalence of classic PTC in our cohort (11/21) is different from the literature (35–37) and may be responsible for the enrichment in BRAF-like mutations in our cohort as compared to RAS-like mutations, thus increasing the specificity of our diagnostic approach. It will be important to test the performance of our algorithm in cohorts characterized by higher prevalence of RAS-like tumors. We are currently conducting a prospective study to obtain validation of this pilot study in an independent cohort with analysis of the assay performance on cytologic specimens, using a new version of Oncomine™ (OCAv3).

## Conclusion

We propose a diagnostic algorhitm utilizing a combination of US features and next generation sequencing that appears to provide a cost-effective diagnostic tool to guide the management strategy of indeterminate thyroid nodules. Our data suggest that molecular testing could be avoided in US high-risk nodules diagnosed in centers with experienced endocrinologists/radiologists evaluating US images, as the specificity of cancer diagnosis in such scenarios is non-inferior to molecular testing. Molecular testing might be beneficial in low-to-intermediate risk sonographic patterns of thyroid nodules as evaluation of genetic landscape in such lesions increases the specificity of cancer diagnosis, and as such, may lead to the avoidance of unnecessary surgeries in these patients.

## Data Availability Statement

The datasets generated for this study can be found in the NCBI BioProject repository, in the following link: https://www.ncbi.nlm.nih.gov/bioproject/PRJNA600873/.

## Ethics Statement

The studies involving human participants were reviewed and approved by NIH Intramural Institutional Review Board. The patients/participants provided their written informed consent to participate in this study.

## Author Contributions

J-KG: conception and design of the study, revision of the final version of the manuscript. CG-L: organization of the database and elaboration of the first draft of the manuscript. SA: statistical analysis. ST: DNA extraction and lab procedures. MZ, CC, and RM: patients recruitment and patients care coordination. AF, MR, SP, and LX: review of pathology material and performance of the molecular testing. LW and KB: revision of the final version of the manuscript. All authors contributed to manuscript revision, read, and approved the submitted version.

### Conflict of Interest

The authors declare that the research was conducted in the absence of any commercial or financial relationships that could be construed as a potential conflict of interest.

## Supplementary Material

Supplemental Figure 1 Oncomine v2 gene list.

Supplemental Figure 2 **(A)** Comparison of diagnostic accuracy of different cut-off points of X_US_. **(B)** Comparison of diagnostic accuracy of different cut-off points of X_GC_. **(C)** Comparison of diagnostic accuracy of different cut-off points of TRS.

Supplemental Figure 3 **(A)** Lack of added benefit of X_GC_ of 0.7 and TRS of 1.6 in thyroid nodules characterized by X_US_ of 0.9 (high-risk US features). TRS is equal 1.6 as all nodules in this group were characterized by X_US_ of 0.9. TRS = X_US_ + X_GC_ = 0.9 + 0.7 = 1.6. **(B)** Diagnostic accuracy of X_US_, X_GC_, and TRS in nodules with X_US_ < 0.9 (low-intermediate risk sonographic features). TRS and X_GC_ are associated with significantly improvement in diagnostic accuracy compared with X_US_ alone.

Supplemental Figure 4 Sub-analysis of the cohort excluding patients with medullary thyroid cancer (MTC)—Predicted performance of each score in populations with different cancer prevalence. Negative predictive value (NPV) in red and positive predictive value (PPV) in blue. **(A)** Ultrasound score (X_US_); **(B)** genomic classifier score (X_GC_); and **(C)** total risk score (TRS).

Supplemental Figure 5 Sub-analysis of the cohort excluding patients with medullary thyroid cancer (MTC)—Comparison of ROC curves-diagnostic accuracy of X_US_, X_GC_, and TRS.

Supplemental Figure 6 Sub-analysis of the cohort excluding patients with medullary thyroid cancer (MTC)—**(A)** Comparison of diagnostic accuracy of different cut-off points of X_US_; **(B)** comparison of diagnostic accuracy of different cut-off points of X_GC_; and **(C)** comparison of diagnostic accuracy of different cut-off pointsof TRS.

## References

[1] Cibas ES Ali SZ. The Bethesda system for reporting thyroid cytopathology. Thyroid. (2009) 19:1159–65. 10.1089/thy.2009.0274 19888858

[2] Bongiovanni M Spitale A Faquin WC Mazzucchelli L Baloch ZW. The Bethesda system for reporting thyroid cytopathology: a meta-analysis. Acta Cytol. (2012) 56:333–9. 10.1159/000339959 22846422

[3] Haugen BR Alexander EK Bible KC Doherty GM Mandel SJ Nikiforov YE. 2015 American thyroid association management guidelines for adult patients with thyroid nodules and differentiated thyroid cancer: the American thyroid association guidelines task force on thyroid nodules and differentiated thyroid cancer. Thyroid. (2016) 26:1–133. 10.1089/thy.2015.0020 26462967 PMC4739132

[4] Russ G Bonnema SJ Erdogan MF Durante C Ngu R Leenhardt L. European thyroid association guidelines for ultrasound malignancy risk stratification of thyroid nodules in adults: the EU-TIRADS. Eur Thyroid J. (2017) 6:225–37. 10.1159/000478927 29167761 PMC5652895

[5] Shin JH Baek JH Chung J Ha EJ Kim JH Lee YH. Ultrasonography diagnosis and imaging-based management of thyroid nodules: revised korean society of thyroid radiology consensus statement and recommendations. Korean J Radiol. (2016) 17:370–95. 10.3348/kjr.2016.17.3.370 27134526 PMC4842857

[6] Tessler FN Middleton WD Grant EG Hoang JK Berland LL Teefey SA. ACR thyroid imaging, reporting and data system (TI-RADS): white paper of the ACR TI-RADS committee. J Am Coll Radiol. (2017) 14:587–95. 10.1016/j.jacr.2017.01.046 28372962

[7] Zhang B Tian J Pei S Chen Y He X Dong Y. Machine learning-assisted system for thyroid nodule diagnosis. Thyroid. (2019) 29:858–67. 10.1089/thy.2018.0380 30929637

[8] Ouyang FS Guo BL Ouyang LZ Liu ZW Lin SJ Meng W. Comparison between linear and nonlinear machine-learning algorithms for the classification of thyroid nodules. Eur J Radiol. (2019) 113:251–7. 10.1016/j.ejrad.2019.02.029 30927956

[9] Sollini M Cozzi L Chiti A Kirienko M. Texture analysis and machine learning to characterize suspected thyroid nodules and differentiated thyroid cancer: where do we stand? Eur J Radiol. (2018) 99:1–8. 10.1016/j.ejrad.2017.12.004 29362138

[10] Burman KD Wartofsky L. Clinical practice. Thyroid nodules. N Engl J Med. (2015) 373:2347–56. 10.1056/NEJMcp1415786 26650154

[11] Klubo-Gwiezdzinska J Wartofsky L. The role of molecular diagnostics in the management of indeterminate thyroid nodules. J Clin Endocrinol Metab. (2018) 103:3507–10. 10.1210/jc.2018-01081 30032182 PMC6456919

[12] Nishino M Nikiforova M. Update on molecular testing for cytologically indeterminate thyroid nodules. Arch Pathol Lab Med. (2018) 142:446–57. 10.5858/arpa.2017-0174-RA 29336606

[13] de Koster EJ de Geus-Oei LF Dekkers OM van Engen-van Grunsven I Hamming J Corssmit EPM. Diagnostic utility of molecular and imaging biomarkers in cytological indeterminate thyroid nodules. Endocr Rev. (2018) 39:154–91. 10.1210/er.2017-00133 29300866

[14] Alexander EK Kennedy GC Baloch ZW Cibas ES Chudova D Diggans J. Preoperative diagnosis of benign thyroid nodules with indeterminate cytology. N Engl J Med. (2012) 367:705–15. 10.1056/NEJMoa1203208 22731672

[15] Nikiforova MN Wald AI Roy S Durso MB Nikiforov YE. Targeted next-generation sequencing panel (ThyroSeq) for detection of mutations in thyroid cancer. J Clin Endocrinol Metab. (2013) 98:E1852–60. 10.1210/jc.2013-2292 23979959 PMC3816258

[16] Nikiforov YE Carty SE Chiosea SI Coyne C Duvvuri U Ferris RL. Highly accurate diagnosis of cancer in thyroid nodules with follicular neoplasm/suspicious for a follicular neoplasm cytology by ThyroSeq v2 next-generation sequencing assay. Cancer. (2014) 120:3627–34. 10.1002/cncr.29038 25209362 PMC7737376

[17] Beaudenon-Huibregtse S Alexander EK Guttler RB Hershman JM Babu V Blevins TC. Centralized molecular testing for oncogenic gene mutations complements the local cytopathologic diagnosis of thyroid nodules. Thyroid. (2014) 24:1479–87. 10.1089/thy.2013.0640 24811481

[18] Labourier E Shifrin A Busseniers AE Lupo MA Manganelli ML Andruss B. Molecular testing for miRNA, mRNA, and DNA on fine-needle aspiration improves the preoperative diagnosis of thyroid nodules with indeterminate cytology. J Clin Endocrinol Metab. (2015) 100:2743–50. 10.1210/jc.2015-1158 25965083 PMC4490308

[19] Gustafson D Tyryshkin K Renwick N. microRNA-guided diagnostics in clinical samples. Best Pract Res Clin Endocrinol Metab. (2016) 30:563–75. 10.1016/j.beem.2016.07.002 27923451

[20] Nikiforova MN Mercurio S Wald AI Barbi de Moura M Callenberg K Santana-Santos L. Analytical performance of the ThyroSeq v3 genomic classifier for cancer diagnosis in thyroid nodules. Cancer. (2018) 124:1682–90. 10.1002/cncr.31245 29345728 PMC5891361

[21] Steward DL Carty SE Sippel RS Yang SP Sosa JA Sipos JA. Performance of a multigene genomic classifier in thyroid nodules with indeterminate cytology: a prospective blinded multicenter study. JAMA Oncol. (2018) 5:204–12. 10.1001/jamaoncol.2018.4616 30419129 PMC6439562

[22] Yoon JH Kwon HJ Kim EK Moon HJ Kwak JY. Subcategorization of atypia of undetermined significance/follicular lesion of undetermined significance (AUS/FLUS): a study applying Thyroid Imaging Reporting and Data System (TIRADS). Clin Endocrinol. (2016) 85:275–82. 10.1111/cen.12987 26639612

[23] Tang AL Falciglia M Yang H Mark JR Steward DL. Validation of American thyroid association ultrasound risk assessment of thyroid nodules selected for ultrasound fine-needle aspiration. Thyroid. (2017) 27:1077–82. 10.1089/thy.2016.0555 28657511

[24] Trimboli P Deandrea M Mormile A Ceriani L Garino F Limone PP. American thyroid association ultrasound system for the initial assessment of thyroid nodules: use in stratifying the risk of malignancy of indeterminate lesions. Head Neck. (2018) 40:722–7. 10.1002/hed.25038 29247582

[25] Valderrabano P McGettigan MJ Lam CA Khazai L Thompson ZJ Chung CH. Thyroid nodules with indeterminate cytology: utility of the American thyroid association sonographic patterns for cancer risk stratification. Thyroid. (2018) 28:1004–12. 10.1089/thy.2018.0085 29848195 PMC6916126

[26] Choi SH Kim EK Kwak JY Kim MJ Son EJ. Interobserver and intraobserver variations in ultrasound assessment of thyroid nodules. Thyroid. (2010) 20:167–72. 10.1089/thy.2008.0354 19725777

[27] Kim HG Kwak JY Kim EK Choi SH Moon HJ. Man to man training: can it help improve the diagnostic performances and interobserver variabilities of thyroid ultrasonography in residents? Eur J Radiol. (2012) 81:e352–6. 10.1016/j.ejrad.2011.11.011 22137098

[28] McIver B Castro MR Morris JC Bernet V Smallridge R Henry M. An independent study of a gene expression classifier (Afirma) in the evaluation of cytologically indeterminate thyroid nodules. J Clin Endocrinol Metab. (2014) 99:4069–77. 10.1210/jc.2013-3584 24780044

[29] Lastra RR Pramick MR Crammer CJ LiVolsi VA Baloch ZW. Implications of a suspicious afirma test result in thyroid fine-needle aspiration cytology: an institutional experience. Cancer Cytopathol. (2014) 122:737–44. 10.1002/cncy.21455 25123499

[30] Krane JF. Lessons from early clinical experience with the Afirma gene expression classifier. Cancer Cytopathol. (2014) 122:715–9. 10.1002/cncy.21472 25123593

[31] Patel KN Angell TE Babiarz J Barth NM Blevins T Duh QY. Performance of a genomic sequencing classifier for the preoperative diagnosis of cytologically indeterminate thyroid nodules. JAMA Surg. (2018) 153:817–24. 10.1001/jamasurg.2018.1153 29799911 PMC6583881

[32] Angell TE Heller HT Cibas ES Barletta JA Kim MI Krane JF. Independent comparison of the afirma genomic sequencing classifier and gene expression classifier for cytologically indeterminate thyroid nodules. Thyroid. (2019) 29:650–6. 10.1089/thy.2018.0726 30803388

[33] Endo M Nabhan F Porter K Roll K Shirley L Azaryan I. Afirma gene sequencing classifier compared to gene expression classifier in indeterminate thyroid nodules. Thyroid. (2019) 29:1115–24. 10.1089/thy.2018.0733 31154940 PMC7141558

[34] Nikiforov YE Seethala RR Tallini G Baloch ZW Basolo F Thompson LDR. Nomenclature revision for encapsulated follicular variant of papillary thyroid carcinoma: a paradigm shift to reduce overtreatment of indolent tumors. JAMA Oncol. (2016) 2:1023–9. 10.1001/jamaoncol.2016.0386 27078145 PMC5539411

[35] Valderrabano P McIver B. Evaluation and management of indeterminate thyroid nodules: the revolution of risk stratification beyond cytological diagnosis. Cancer Control. (2017) 24:1073274817729231. 10.1177/1073274817729231 28975825 PMC5937245

[36] Faquin WC Baloch ZW. Fine-needle aspiration of follicular patterned lesions of the thyroid: diagnosis, management, and follow-up according to National Cancer Institute (NCI) recommendations. Diagn Cytopathol. (2010) 38:731–9. 10.1002/dc.21292 20049964

[37] Rago T Scutari M Latrofa F Loiacono V Piaggi P Marchetti I. The large majority of 1520 patients with indeterminate thyroid nodule at cytology have a favorable outcome, and a clinical risk score has a high negative predictive value for a more cumbersome cancer disease. J Clini Endocrinol Metab. (2014) 99:3700–7. 10.1210/jc.2013-4401 24708101

