# Supplementary figures and images for: A Novel Risk Stratification System for Thyroid Nodules With Indeterminate Cytology—A Pilot Cohort Study
Source: Front Endocrinol (Lausanne). 2020 Feb 18;11:53. doi: 10.3389/fendo.2020.00053 (PMC7040241; doi:10.3389/fendo.2020.00053)

## Slide 1
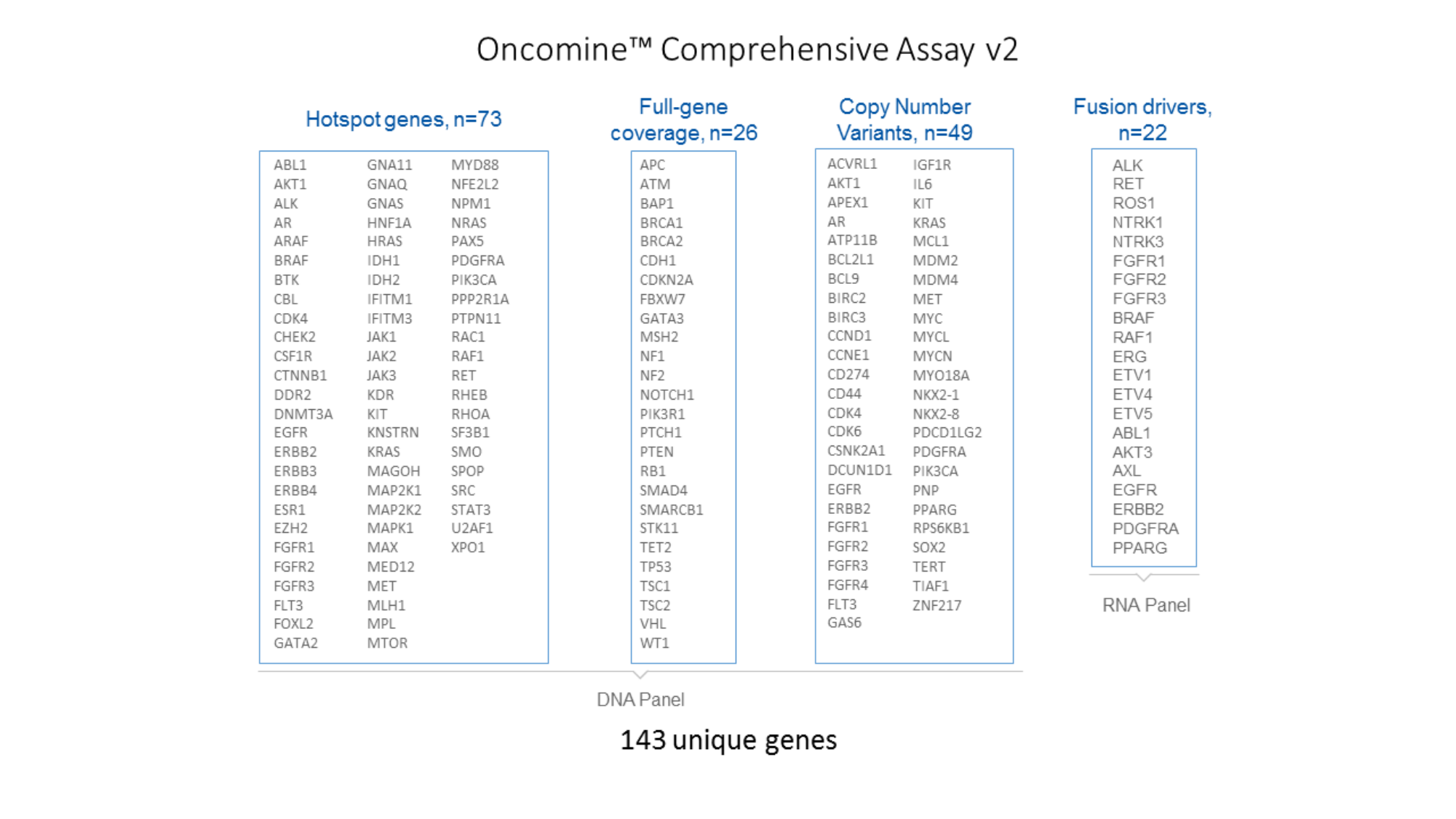

## Slide 2
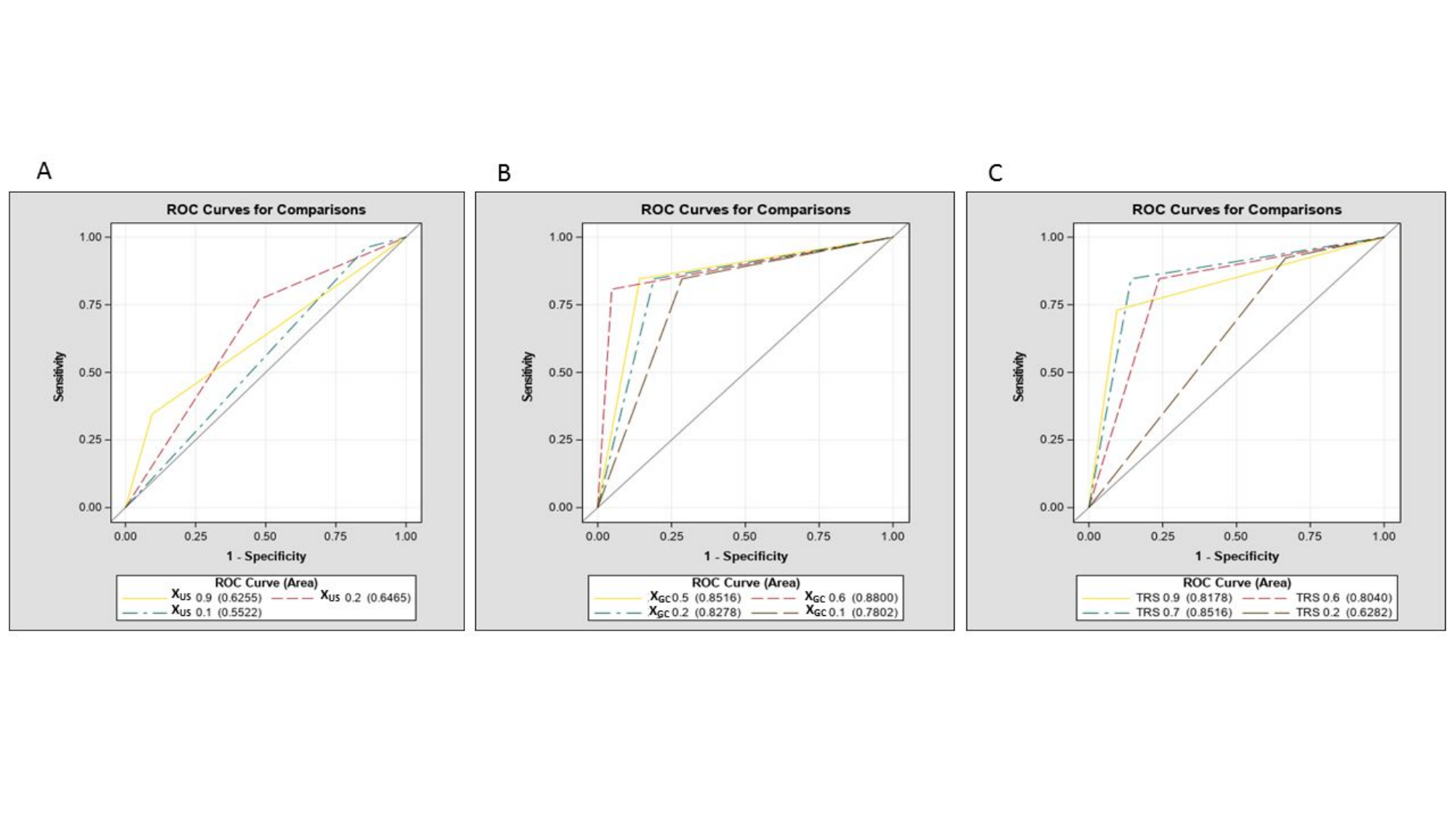

## Slide 3
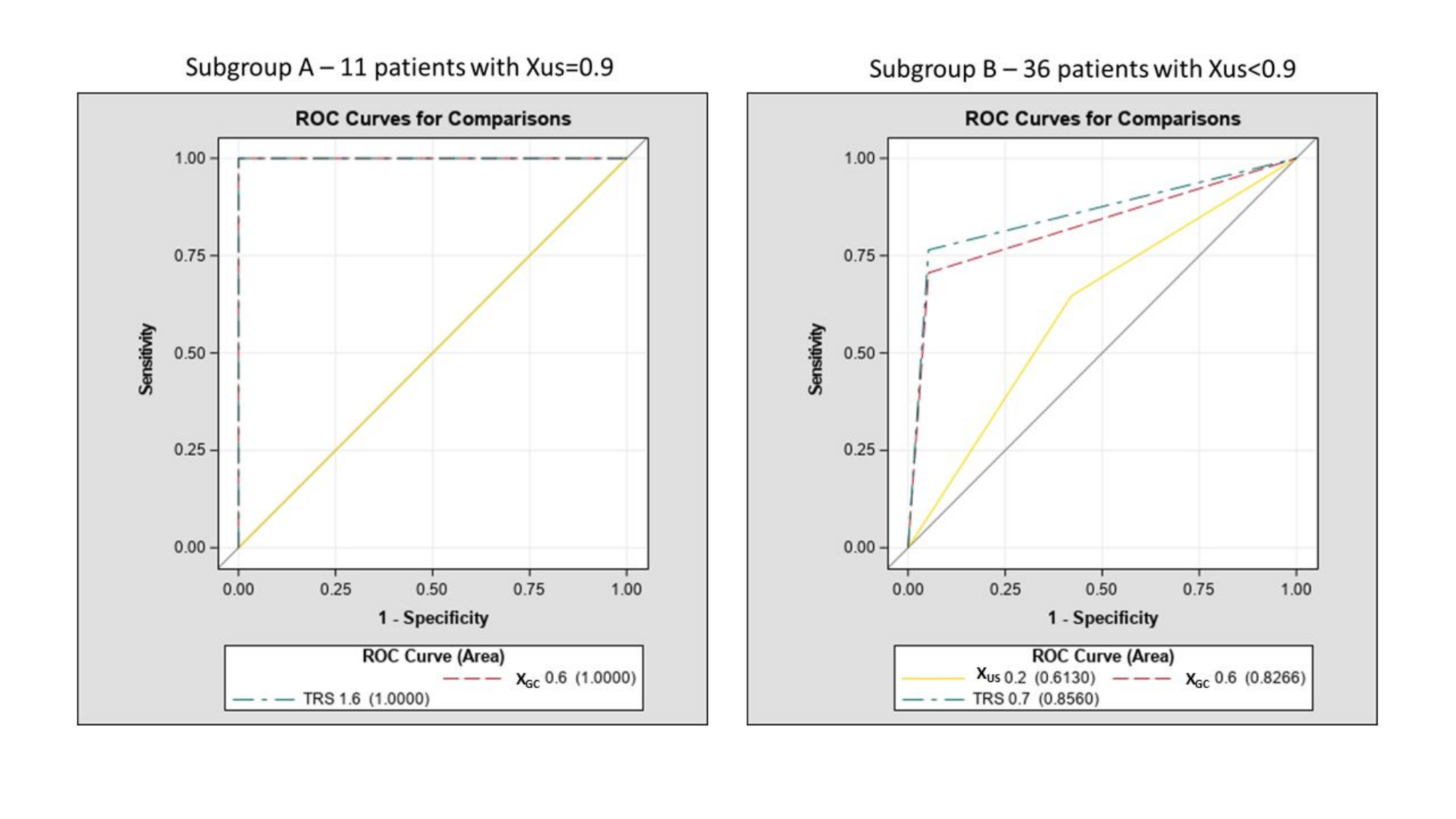

## Slide 4
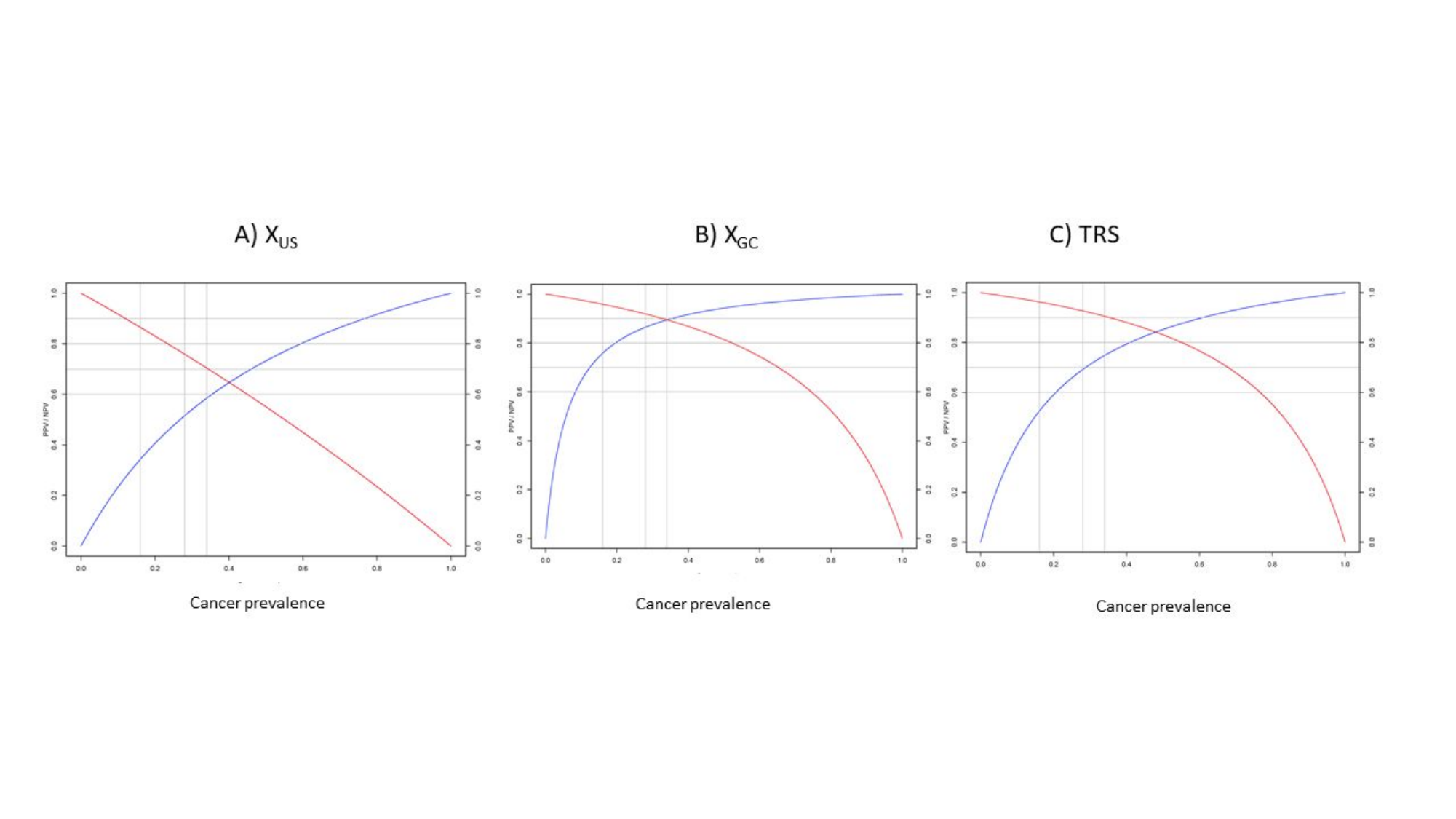

## Slide 5
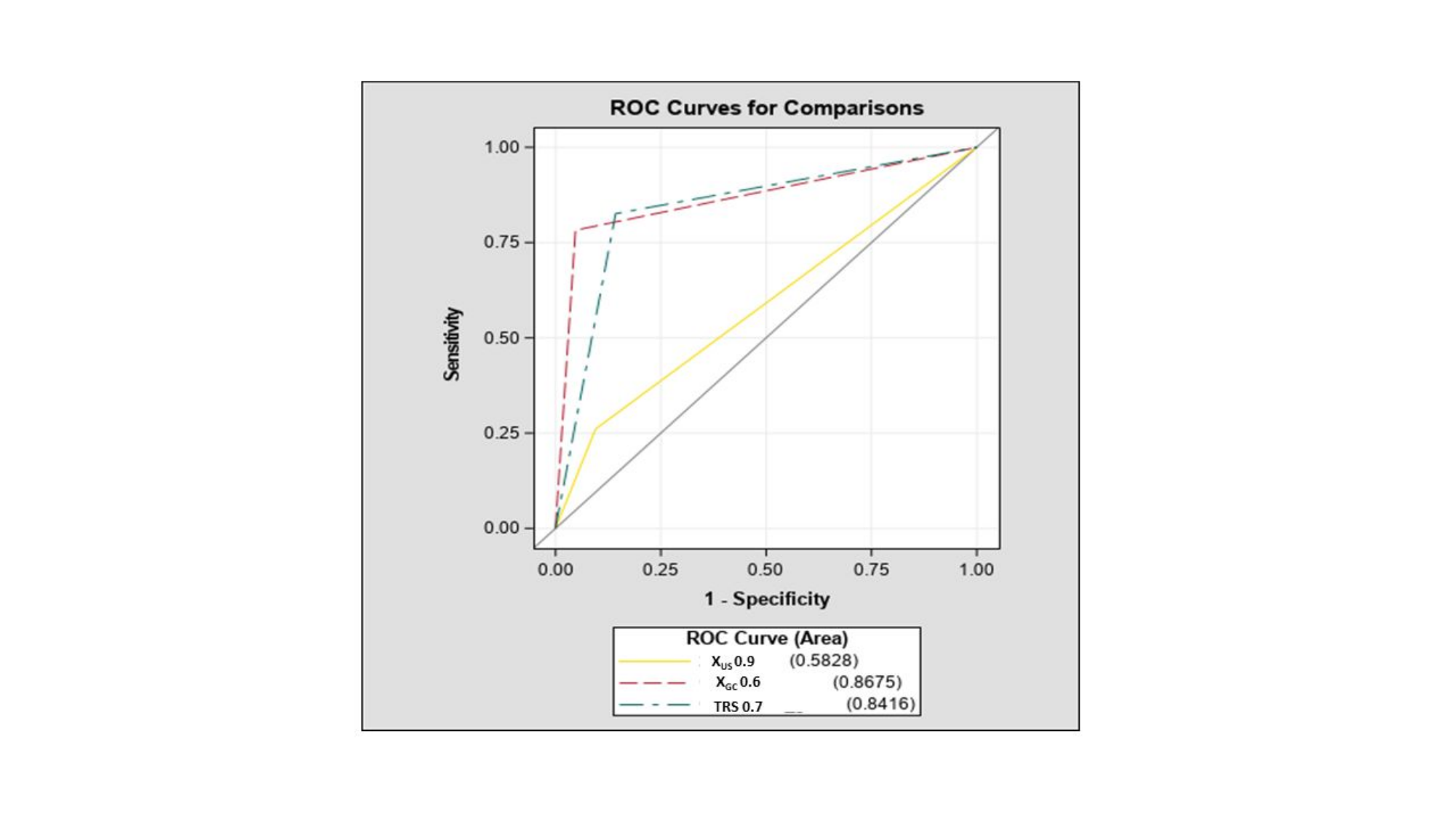

## Slide 6
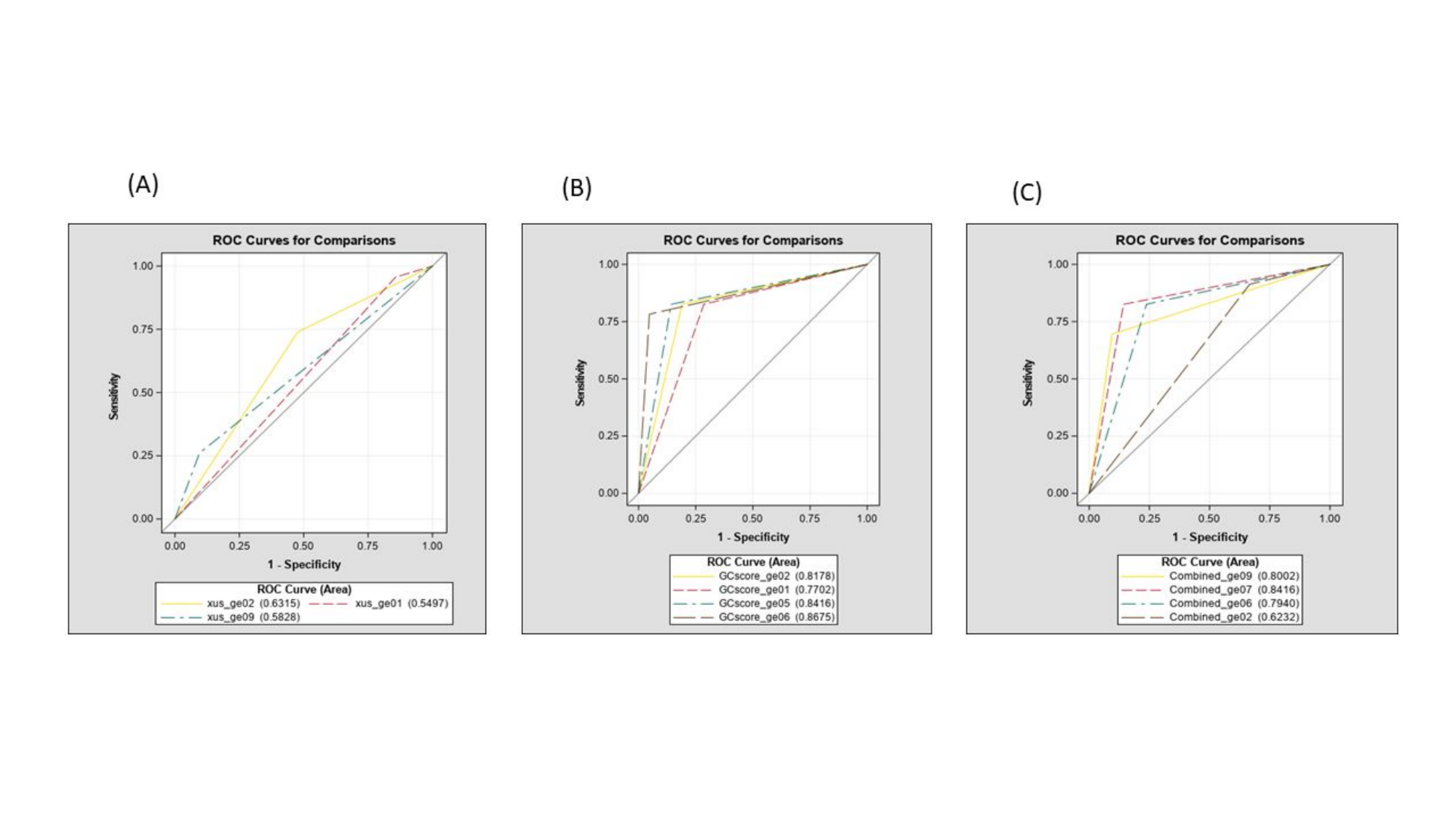

Supplement: Supplemental Figure 1 — Oncomine v2 gene list. [file Presentation_1.pptx]
